# Spreading of *cfr*-Carrying Plasmids among Staphylococci from Humans and Animals

**DOI:** 10.1128/spectrum.02461-22

**Published:** 2022-11-22

**Authors:** Yuan Gao, Zheng Wang, Jiani Fu, Jiachang Cai, Tengfei Ma, Ning Xie, Run Fan, Weishuai Zhai, Andrea T. Feßler, Chengtao Sun, Congming Wu, Stefan Schwarz, Rong Zhang, Yang Wang

**Affiliations:** a Key Laboratory of Animal Antimicrobial Resistance Surveillance, Ministry of Agriculture and Rural Affairs, Beijing, China; b Beijing Key Laboratory of Detection Technology for Animal-Derived Food Safety, College of Veterinary Medicine, China Agricultural University, Beijing, China; c College of Biology and Agricultural Resources, Huanggang Normal University, Huanggang, China; d Second Affiliated Hospital of Zhejiang University, Zhejiang University, Hangzhou, China; e Beijing Integrity Technology Co., Ltd., Beijing, China; f Institute of Microbiology and Epizootics, Centre for Infection Medicine, Department of Veterinary Medicine, Freie Universität Berlin, Berlin, Germany; g Veterinary Centre for Resistance Research, Department of Veterinary Medicine, Freie Universität Berlin, Berlin, Germany; University of Greifswald

**Keywords:** linezolid resistance, *Staphylococcus*, *cfr*, plasmids, conjugative transfer

## Abstract

The multidrug resistance gene *cfr* mediates resistance to multiple antimicrobial agents, including linezolid. Plasmids are the preferred vector for the dissemination of *cfr*. However, the presence and transmission of *cfr*-carrying plasmids among staphylococci from humans and animals have rarely been studied. Here, we investigated the presence of the *cfr* gene in 2,250 staphylococci of human clinical origin collected in Zhejiang, China, in 1998 to 2021 and in 3,329 porcine staphylococci preserved in our laboratories. The *cfr* gene was detected in 38 human isolates; its presence in Staphylococcus haemolyticus and Staphylococcus cohnii in 2003 was earlier than that identified in 2005, and Staphylococcus capitis (*n* = 30) was the predominant species. The *cfr*-carrying fragment in 38 isolates exhibited >99% nucleotide sequence similarity to plasmid pLRSA417 (39,504 bp), which was identified in 2015 and originated from a human clinical methicillin-resistant Staphylococcus aureus isolate from Zhejiang, China. The *cfr*-carrying plasmids in 18 MinION-sequenced staphylococci ranged in size from 32,697 bp to 43,457 bp. Fifteen plasmids were identical to pLRSA417, except for the inversion of an 8.4-kb segment comprising IS*256*-*aacA/aphD*-IS*Enfa4*_1-*cfr*-IS*Enfa4*_2, while the remaining 3 plasmids exhibited slightly different structures. Among the 114 *cfr*-positive staphylococci from pigs, pLRSA417-like plasmids were detected in 3 isolates. Intraspecies and interspecies conjugation occurred in human-derived pLRSA417-like plasmids. The presence of pLRSA417-like plasmids in staphylococci from multiple geographic regions and different hosts implied the possible transmission of the respective isolates between humans and animals.

**IMPORTANCE** The therapeutic efficacy of the oxazolidinone antimicrobial linezolid is reduced by the emergence and dissemination of the multidrug resistance gene *cfr*. The *cfr*-carrying plasmid pLRSA417 was first identified in a clinical methicillin-resistant Staphylococcus aureus isolate, but its presence in staphylococci of human and animal origin has not been reported previously. This study showed that conjugative plasmids similar to pLRSA417 were detected mainly in Staphylococcus capitis and existed in different staphylococci in 2003 to 2021 in various clinical departments in the same hospital. pLRSA417-like plasmids were also present in staphylococci of food animal sources from different geographic regions, which suggested possible transmission among humans and animals.

## INTRODUCTION

The oxazolidinone linezolid is preferentially used for the treatment of diseases, such as skin infections and bloodstream infections, caused by multidrug-resistant Staphylococcus aureus (1, 2). However, the emergence of linezolid resistance genes, such as *cfr*, has impaired the treatment efficacy of oxazolidinones (3). The gene *cfr* encodes an RNA methyltransferase that mediates resistance against phenicols, lincosamides, oxazolidinones, pleuromutilins, and streptogramin A antimicrobial agents (4, 5). During the past 2 decades, this gene has been disseminated globally in bacteria of human and animal origin (6–9). Mobile genetic elements play a crucial role in the dissemination of *cfr*, among which plasmids are the preferred vectors (7, 8).

Numerous *cfr*-carrying plasmids have been identified in staphylococci, enterococci, and other bacteria (8), such as the well-characterized plasmids pSCFS7 (10), pERGB (11), and p12-00322 (12), among others. Plasmid pLRSA417 is one of the *cfr*-carrying plasmids identified from a human clinical methicillin-resistant S. aureus (MRSA) isolate in Zhejiang, China, in 2015 (13). However, the prevalence of pLRSA417-like plasmids in bacteria of human and animal origin has not been demonstrated previously. In this study, we identified pLRSA417-like plasmids in staphylococci isolated from human infections since 2003 and from nasal or skin samples collected from healthy pigs in 2015 and 2016, and we showed that these plasmids can spread via conjugation.

## RESULTS

### Bacterial isolation and identification of *cfr*-carrying plasmids among human staphylococci.

A total of 2,250 Staphylococcus isolates, including 1,141 S. aureus isolates and 1,109 coagulase-negative staphylococci (CoNS) isolates, were obtained from clinical samples from different departments of a hospital in Hangzhou, Zhejiang, China, during the period from 1998 to 2014 (see Table S1 in the supplemental material). Among these isolates, 34 (1.51%) *cfr*-positive Staphylococcus isolates were obtained from the intensive care unit (*n* = 21), burns department (*n* = 5), emergency intensive care unit (*n* = 2), respiratory medicine department (*n* = 2), emergency medicine department (*n* = 2), urology surgery (*n* = 1), and infectious diseases department (*n* = 1) (see Table S2). CoNS accounted for the majority of the *cfr*-positive isolates (*n* = 33), including 26 Staphylococcus capitis isolates, 4 Staphylococcus haemolyticus isolates, and 3 Staphylococcus cohnii isolates; the remaining isolate was S. aureus. Notably, the *cfr* gene was detected in a S. haemolyticus isolate and a S. cohnii isolate identified in 2003. Two staphylococci, the S. capitis isolate TZ266 and the S. cohnii isolate TZ273, were obtained from the same blood sample from a patient in the intensive care unit. In addition, 4 *cfr*-positive S. capitis isolates were obtained from the burns department in this hospital in 2019 to 2021, but unfortunately the background information for these isolates was not available.

Whole-genome sequencing (WGS) analysis with an Illumina HiSeq system and BLAST Ring Image Generator (BRIG) analysis revealed that the *cfr*-carrying fragment in all 38 isolates exhibited >99% nucleotide sequence identity to the corresponding part of plasmid pLRSA417 (GenBank accession number KJ922127.1) (39,504 bp), which was identified in a clinical linezolid-resistant MRSA isolate from Zhejiang, China, in 2013 (see Fig. S1).

### Sequence analysis of *cfr*-carrying plasmids in staphylococci from humans and animals.

**FIG 1:**
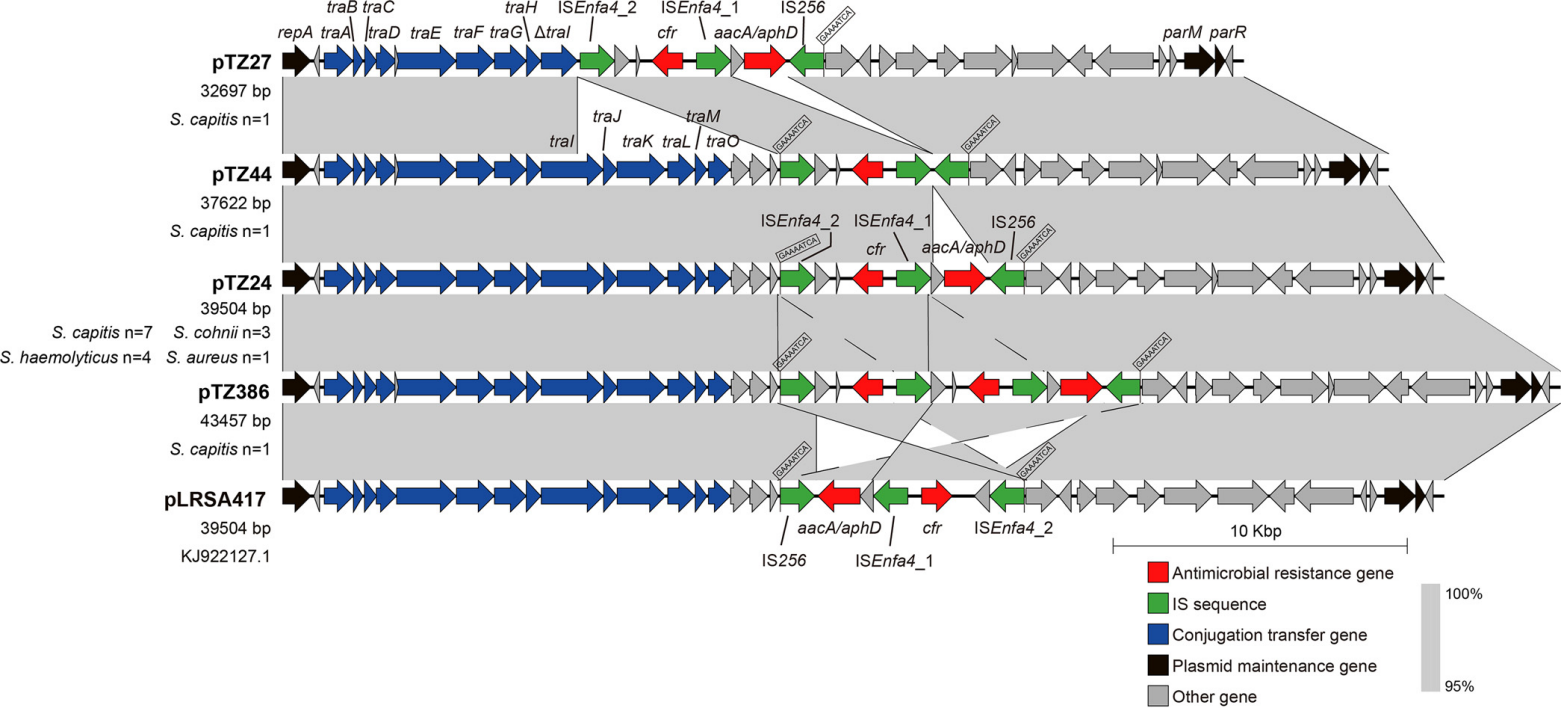
Sequence alignments between plasmid pLRSA417 and *cfr*-carrying plasmids from staphylococci of human origin identified in this study. The gray shading indicates the homologous regions. Arrows in different colors indicate different types of genes. The target duplication sequences caused by IS sequences are indicated.

To verify the location of the *cfr*-carrying fragments, 18 *cfr*-positive staphylococci were chosen, according to the selection criteria detailed in Materials and Methods, for third-generation sequencing. The sequence data revealed that all *cfr* genes were located on plasmids ranging in size from 32,679 bp to 43,457 bp (Table 1). All of these plasmids showed high homology (query coverage, 100%; identity, >99%) with pLRSA417, despite variations in their sizes (Fig. 1 and Table 1). These plasmids included 3 S. capitis plasmids of 32,697 bp (pTZ27), 37,622 bp (pTZ44), and 43,457 bp (pTZ386), as well as 14 same-sized plasmids of 39,504 bp and 1 plasmid (pTZ361) of 39,503 bp. These 39,504-bp plasmids exhibited >99% nucleotide sequence identity to pLRSA417 but differed from pLRSA417 by the inversion of an 8.4-kb fragment containing *cfr*. This fragment was identical (99.53%) to that of plasmid pSS-01 from a porcine S. cohnii isolate (GenBank accession number JF834909.1) and comprised IS*256*-*aacA/aphD*-IS*Enfa4*_1-*cfr*-IS*Enfa4*_2 flanked by 8-bp target site duplications (5′-GAAAATCA-3′) (Fig. 1). The S. haemolyticus plasmid pTZ361 (39,503 bp) exhibited a 1-bp deletion within the IS*256* element, which resulted in a frameshift of the transposase gene that generated a premature stop codon (288 amino acids versus 390 amino acids). The 32,697-bp plasmid pTZ27 showed a deletion of a 6,807-bp segment carrying six genes involved in conjugative transfer (*traI* [truncated], *traJ*, *traK*, *traL*, *traM*, and *traO*) and two *hp* genes for hypothetical proteins. In the 37,622-bp plasmid pTZ44, the aminoglycoside resistance gene *aacA/aphD* and the *hp* gene between IS*256* and IS*Enfa4*_1 were missing. A duplication of the segment IS*Enfa4*_2-*hp*-*cfr* was observed in the 43,457-bp plasmid pTZ386.

**TABLE 1:** Plasmids carrying *cfr* from human Staphylococcus spp. identified in this study

Plasmid	Species	Year of isolation	Size (bp)
pTZ361	S. haemolyticus	2003	39,503
pTZ360	S. cohnii	2003	39,504
pTZ390_1	S. haemolyticus	2004	39,504
pTZ386	S. capitis	2004	43,457
pTZ24	S. capitis	2011	39,504
pTZ14	S. cohnii	2011	39,504
pTZ41	S. aureus	2011	39,504
pTZ100	S. capitis	2012	39,504
pTZ266	S. capitis	2012	39,504
pTZ99	S. haemolyticus	2012	39,504
pTZ273	S. cohnii	2012	39,504
pTZ390_2	S. capitis	2013	39,504
pTZ477	S. haemolyticus	2013	39,504
pTZ931	S. capitis	2014	39,504
pTZ10	S. capitis	2019	39,504
pTZ27	S. capitis	2020	32,697
pTZ44	S. capitis	2021	37,622
pTZ47	S. capitis	2021	39,504

**FIG 2:**
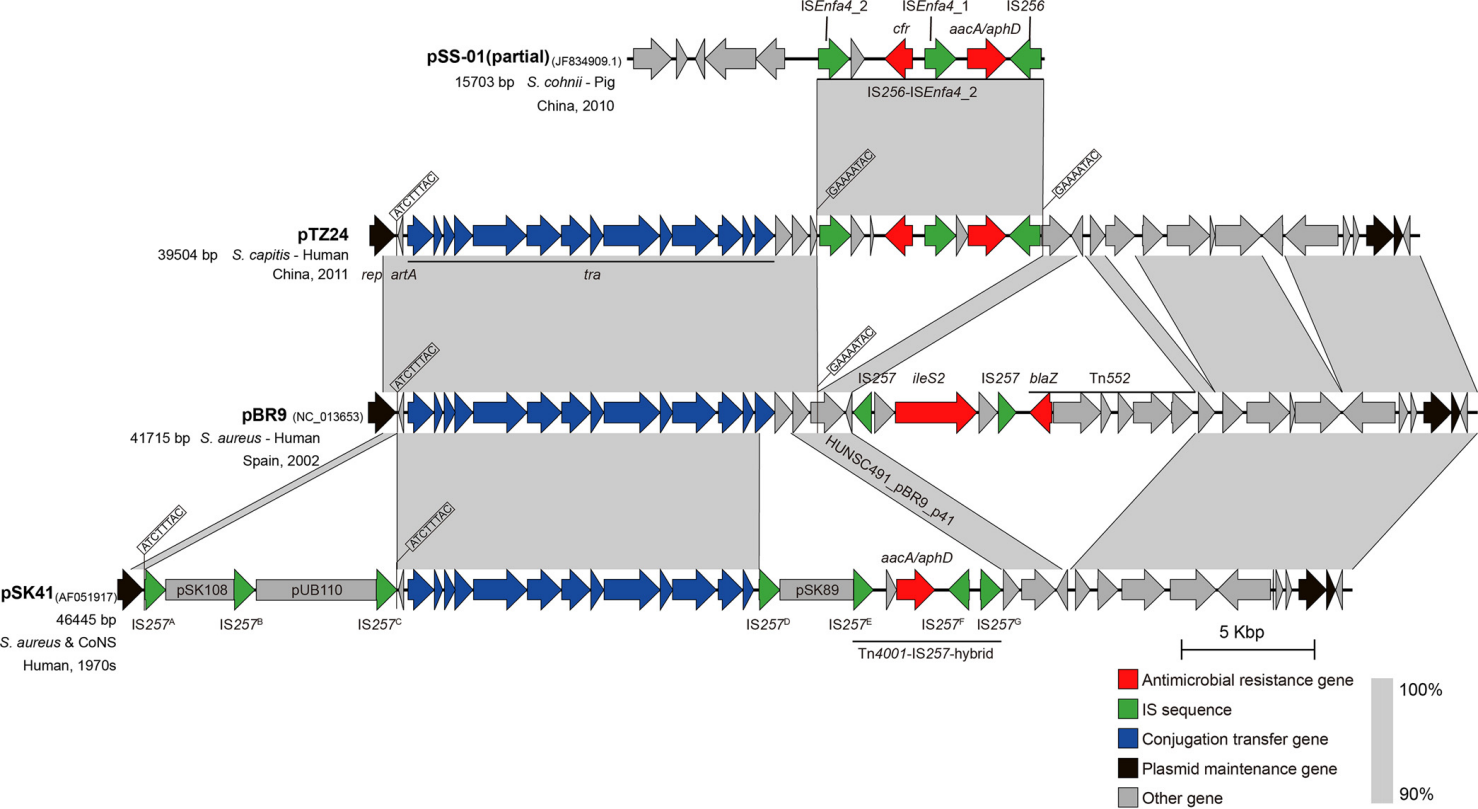
Sequence alignments between the plasmids pSS-01, pTZ24, pBR9, and pSK41. The gray shading indicates the homologous regions. Arrows in different colors indicate different types of genes. The plasmid names, GenBank accession numbers, sizes, sources, isolation regions, and isolation times are shown on the left. The target duplication sequences and integrated RCR plasmids are indicated.

All pLRSA417-like plasmids showed >94% nucleotide sequence identity with the human-derived conjugative multidrug resistance plasmid pBR9 (GenBank accession number NC_013653), belonging to the pSK41 family (Fig. 2) (14). The IS*256*-IS*Enfa4*_2 fragment containing *cfr* was integrated into the pBR9 backbone at the GAAAATCA site within the hypothetical protein gene HUNSC491_pPR9_p41. However, pBR9 differed in its resistance gene content from the pLRSA417-like plasmids identified in this study. A Tn*552*-like transposon and an IS*257*-*ileS2*-IS*257* fragment, carrying the β-lactamase gene *blaZ* and the mupirocin-resistance gene *ileS2*, respectively, were integrated downstream of the IS*256*-IS*Enfa4*_2 fragment in plasmid pBR9.

**FIG 3:**
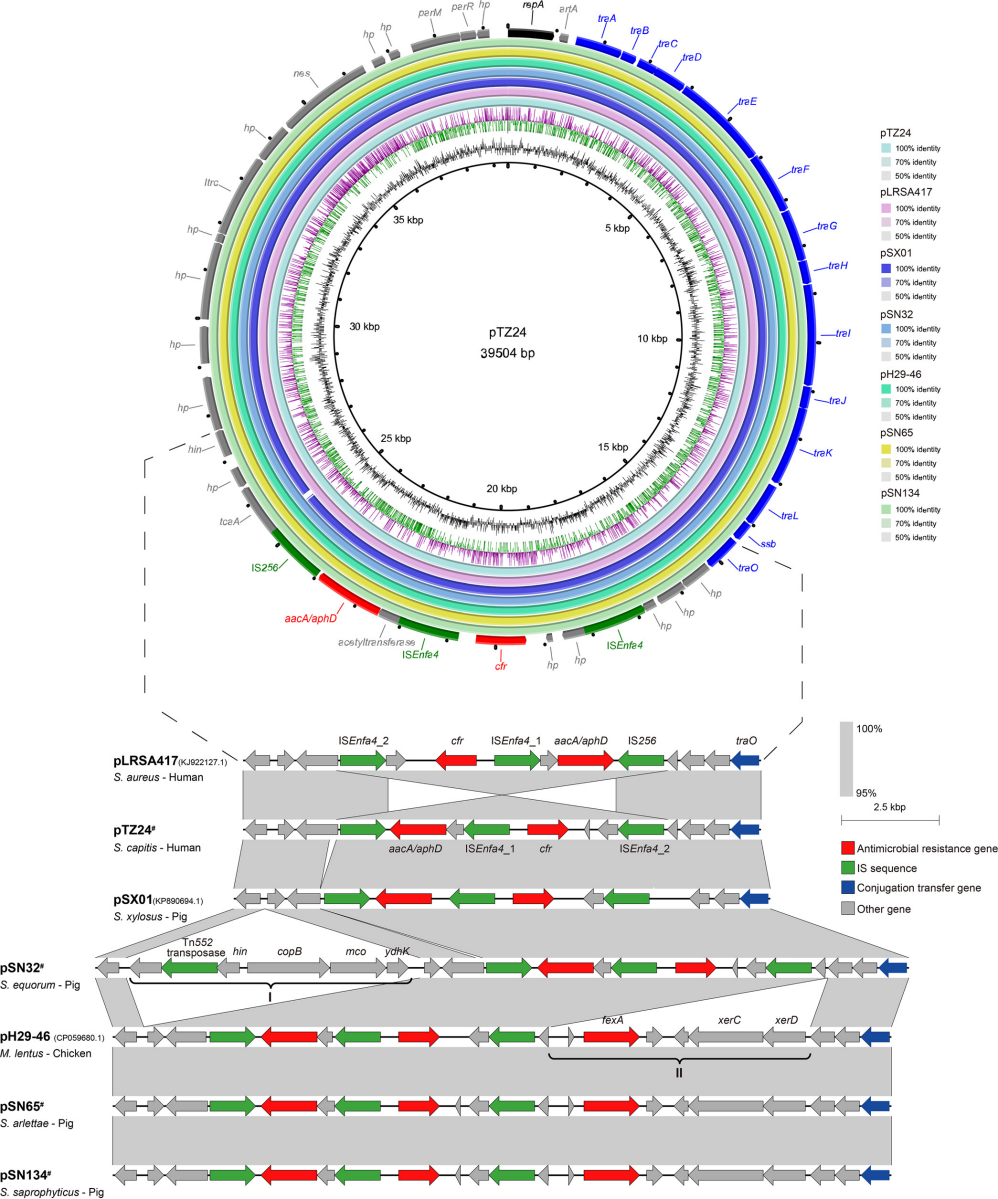
Sequence alignments of pLRSA417-like plasmids from human clinical staphylococci and animal staphylococci. Different plasmids are displayed in different colors (the innermost rings shows the GC content [black] and GC skew [purple/olive]), and the outermost ring shows the functional genes. Shades of gray indicate the homologous segments. Arrows in different colors indicate different genes. #, Plasmids identified in this study.

Because the pLRSA417-like plasmids were predominant among the *cfr*-carrying staphylococci of human origin, we further investigated their presence in staphylococci of animal origin. The Illumina HiSeq sequences of a total of 114 *cfr*-positive isolates of 3,329 porcine staphylococci collected from Shanghai, Guangdong, Shandong, and Henan provinces in 2008 to 2016 were screened for the presence of pLRSA417-like plasmids (15–18). Two Staphylococcus equorum isolates, SN32 and SN65, from nasal swab samples collected in Henan in 2016 and a Staphylococcus saprophyticus isolate, SN134, from a skin swab sample collected in Shandong in 2015 (see Table S3) were positive for pLRSA417-like *cfr*-carrying plasmids (designated pSN32, pSN65, and pSN134, respectively) and were further analyzed by third-generation sequencing. Moreover, 8 *cfr*-carrying pLRSA417-like plasmids (>95% nucleotide sequence identity), all originating from staphylococci, were obtained from the NCBI GenBank database (see Table S4). Two representative plasmids, namely, pSX01 (GenBank accession number KP890694.1) from a porcine Staphylococcus xylosus isolate from Henan and pH29-46 (GenBank accession number CP059680.1) from a Mammaliicoccus (formerly Staphylococcus) lentus isolate of chicken origin from Zhejiang, were used for comparative analysis in combination with plasmids pLRSA417, pTZ24, pSN32, pSN65, and pSN134. The backbones were identical among all 7 plasmids, while the inversion, insertion, or deletion events occurred near the IS-rich region (Fig. 3). Compared with pLRSA417, the inversion of the 8.4-kb segment also occurred in all 5 plasmids of animal origin. The 47,066-bp plasmid pSN32 exhibited a 7,528-bp insertion identical (99.81% nucleotide sequence identity) to that in the Mammaliicoccus (formerly Staphylococcus) *sciuri* Wo19-3 plasmid-like sequence (GenBank accession number KX982172.1) (see Fig. S2A), consisting of a Tn*552* transposase gene, an AAA family ATPase gene, a DNA-invertase gene *hin*, a copper-exporting P-type ATPase B gene *copB*, a multicopper oxidase gene *mco*, and two *hp* genes (Fig. 3, region I). The 46,169-bp plasmids pSN65 and pSN134 and the 46,167-bp plasmid pH29-46 showed high homology to one another, with only 5 to 11 single-nucleotide polymorphisms (SNPs). They all carried an additional 6,640-bp region identical (99.97% identity) to that in the S. saprophyticus plasmid pY8P168P-cfr (GenBank accession number CP065798.1) (see Fig. S2B), containing the florfenicol resistance gene *fexA* and the tyrosine recombinase genes *xerC* and *xerD* near IS*256* (Fig. 3, region II). In addition, the pig-derived 39,969-bp plasmid pSX01 showed high similarity to the 39,504-bp plasmid pTZ24 of human origin (query coverage, 99%; identity, 99.96%).

### Conjugative transfer of *cfr*-carrying plasmids.

It is noteworthy that pLRSA417-like plasmids were found in several human clinical Staphylococcus species and were mainly prevalent in S. capitis. In addition, the identical plasmids pTZ266 from S. capitis TZ266 and pTZ273 from S. cohnii TZ273 originated from the same patient, indicating that pLRSA417-like plasmids were capable of interspecies dissemination through horizontal transfer even *in vivo*. Filter mating assays served to prove the conjugative transfer ability of pLRSA417-like plasmids. S. capitis plasmid pTZ24, S. haemolyticus plasmid pTZ99, S. cohnii plasmid pTZ273, and S. aureus plasmid pTZ41 could transfer from human clinical isolates to S. aureus RN4220NR and Enterococcus faecalis JH2-2 recipient strains. Moreover, the transconjugants described above, namely, RN4220-*cfr* and JH2-2-*cfr*, were used as donor strains, and S. capitis TZ24, which lost the *cfr*-carrying plasmid during serial passage (designated TZ24′), was chosen as the recipient strain. A transfer frequency of nearly 10^−3^ transconjugants obtained per recipient was observed. These results indicated the intraspecies and interspecies conjugation ability of these plasmids and the ability of S. capitis TZ24′ to acquire pLRSA417-like plasmids.

## DISCUSSION

The multiresistance gene *cfr* was first identified in a bovine M. sciuri isolate from Germany in 2000, and the first human isolate carrying the *cfr* gene was a clinical MRSA isolate from Colombia in 2005 (3, 19). Here, we observed 2 *cfr*-carrying CoNS isolates from human clinical infections identified in 2003, indicating that the emergence of *cfr* in clinics in China was earlier than reported previously. To the best of our knowledge, this is the first report of pLRSA417-like plasmids detected in CoNS species, including S. haemolyticus, S. cohnii, S. equorum, Staphylococcu arlettae, and S. saprophyticus. In addition, the S. capitis and other CoNS species accounted for the majority of *cfr*-positive isolates (37/38 isolates [97.37%]), indicating the preference of *cfr* for CoNS rather than S. aureus.

Both plasmid-borne and chromosome-borne *cfr* genes were detected in Staphylococcus spp. of various origins (7–9). Based on the staphylococcal *cfr*-containing sequences in the NCBI GenBank database, the *cfr* gene was present on plasmids in the majority of staphylococci of both human (27/32 isolates) and animal (30/38 isolates) origin (see Table S5 in the supplemental material). This observation indicated the critical role of plasmids for the dissemination of the *cfr* gene. The *cfr* gene was frequently bracketed by IS sequences in the same orientation (8), which may form translocatable units and support its transfer via translocation between plasmids while also supporting its integration into chromosomal DNA (7, 8). The high mobility of the *cfr* gene, together with the high levels of similarity of pLRSA417-like plasmids present in staphylococci from both humans and animals, and the higher rates of detection of *cfr* in animal isolates (3.42% [114/3,329 isolates]) than in human clinical isolates (1.51% [34/2,250 isolates]) support the assumption that humans may acquire *cfr*-positive Staphylococcus isolates through contact with animals (20). Continuous surveillance of *cfr*-positive bacteria is needed to verify and monitor the routes of transmission of the *cfr* gene between humans and animals.

In this study, we identified pLRSA417-like plasmids in clinical S. haemolyticus and S. cohnii isolates from as early as 2003. The pLRSA417-like plasmids, represented by pTZ24 in this study, shared high sequence similarity with the human-derived plasmid pBR9, belonging to the pSK41 family. Plasmid pSK41 is a prototype multidrug resistance plasmid from S. aureus (14, 21). The sequence of pSK41 comprised seven IS*257* copies (A to G), via which small rolling-circle replicating (RCR) plasmids became integrated, i.e., pSK108 (IS*257*^A^-IS*257*^B^), pUB110 (IS*257*^B^-IS*257*^C^), pSK89 (IS*257*^D^-IS*257*^E^), and an insertion fragment Tn*4001*-IS*257* hybrid structure (IS*257*^E^-IS*257*^G^). The integrated RCR plasmids (pSK108, pUB110 and pSK89) were missing in both plasmid pTZ24 and plasmid pBR9, but all three plasmids shared large parts of the transfer (*tra*) gene region, as well as cargo genes, including those required for plasmid maintenance. Plasmids pTZ24 and pBR9 were more closely related, and it is likely that they developed from the same ancestor plasmid. Because both plasmids differed distinctly in their resistance genes and the resistance genes were flanked by insertion sequences, this observation suggests divergent development of these plasmids from a basic plasmid type into novel plasmids in which the resistance-gene-carrying segments were incorporated via insertion sequences.

Since the first identification of plasmid pLRSA417 in 2003, similar plasmids were identified in different Staphylococcus species from different wards of the same hospital until 2021, which implies the high horizonal transfer ability of pLRSA417-like plasmids among clinical staphylococci. This horizonal transfer ability of *cfr*-carrying plasmids was confirmed in our study by proving that pLRSA417-like plasmids could transfer from human staphylococci into the recipient strains S. aureus RN4220NR and E. faecalis JH2-2 via conjugation. Similar phenomena were observed for other *cfr*-carrying plasmids such as p12-00322 from Staphylococcus epidermidis, which shared 79% coverage (including conjugative transfer genes) and 96.97% identity with pLRSA417 (12). In addition, pLRSA417-like plasmids could transfer back into their original plasmid-cured host strain at a relatively high frequency. Furthermore, S. capitis accounted for the majority (78.9% [30/38 isolates]) of human *cfr*-positive staphylococci, and WGS of the isolates indicated the preferential association of S. capitis isolates (55.6% [10/18 isolates]) with pLRSA417-like plasmids. Hence, systematic monitoring of pLRSA417-like plasmids, especially in linezolid-resistant CoNS such as S. capitis, is needed.

The identification of pLRSA417-like plasmids in staphylococci from human and animal sources implies the possibility of plasmid transfer between human and animal isolates. In examining the presence of pLRSA417-like plasmids in the NCBI GenBank database, these plasmids were found in various Staphylococcus species from both Asia and Europe, including human clinical isolates as well as isolates from livestock (see Table S4). S. aureus and some CoNS, such as S. capitis and S. haemolyticus, may cause infections in both humans and animals (22, 23). The *cfr*-carrying plasmid in animal staphylococci may disseminate into human-derived staphylococci through the food chain or environment under the selection pressure provided by veterinary antimicrobial agents, including florfenicol (phenicols), lincomycin and clindamycin (lincosamides), and tiamulin and valnemulin (pleuromutilins). Prudent use and risk assessment of these antimicrobials in veterinary medicine are needed.

In conclusion, this study described a number of *cfr*-carrying plasmids related to pLRSA417 in Staphylococcus species of human clinical origin in Zhejiang, China, obtained as early as 2003. These pLRSA417-like plasmids were also identified in Staphylococcus species of pig origin. The abilities for *in vitro* intraspecies and interspecies transfer of pLRSA417-like plasmids were confirmed. The possible transmission of pLRSA417-like plasmids between staphylococci from humans and animals and the spreading and persistence of pLRSA417-like-plasmid-carrying S. capitis strains in human clinics require substantial attention.

## MATERIALS AND METHODS

### Bacterial isolation.

A total of 2,250 clinical Staphylococcus sp. isolates collected from a hospital in Hangzhou, Zhejiang, China, in 1998 to 2014 were screened for their growth on brain heart infusion (BHI) agar containing 10 mg/L florfenicol. The bacterial species was identified using matrix-assisted laser desorption ionization–time of flight (MALDI-TOF) mass spectrometry (Bruker, Germany), and the presence of *cfr* was confirmed by PCR and Sanger sequencing (24). Four *cfr*-positive S. capitis isolates obtained from the burns department in 2019 to 2021 were confirmed through the aforementioned methods.

### DNA extraction, WGS, and sequence alignment.

Genomic DNA of *cfr*-positive Staphylococcus isolates was extracted with the TIANamp bacteria DNA kit (Tiangen, Beijing, China) and sequenced (paired-end 150-bp reads) using the Illumina HiSeq platform (Personalbio, Shanghai, China). The sequences were assembled using SPAdes v3.9.0. Furthermore, 18 *cfr*-positive Staphylococcus isolates from humans were selected (all of the isolates were chosen when the number of isolates of a certain staphylococcal species was <10, and one-third of the isolates were randomly selected when the corresponding number of isolates was >10), additionally sequenced with the Oxford Nanopore Technologies MinION platform, and assembled using hybrid Illumina-Nanopore assembly via Unicycler v0.4.8. The sequences of human staphylococci were used as references to analyze porcine staphylococci preserved in our laboratories. Based on the Illumina HiSeq sequences of 114 porcine *cfr*-positive staphylococci, 3 Staphylococcus isolates whose *cfr*-flanking sequences were similar to those of the human isolates were selected for sequencing with an Oxford Nanopore Technologies MinION system. Prokka v1.14.6 was used to annotate the assembled genomes. Sequence alignments via NCBI BLASTn (https://blast.ncbi.nlm.nih.gov/Blast.cgi) served for analysis of the *cfr*-carrying plasmids. Visualization of sequence comparisons was accomplished via BRIG (25) and Easyfig (26).

### Filter mating experiments.

The transfer frequency of plasmids was investigated by filter mating experiments as described previously (27). S. capitis TZ24, S. haemolyticus TZ99, S. cohnii TZ273, and S. aureus TZ41 were selected to represent the species identified in this study and served as the donor strains, whereas S. aureus RN4220NR (28) and E. faecalis JH2-2 were chosen as the recipient strains. Transconjugants were selected on BHI agar plates supplemented with florfenicol (10 mg/L) and rifampicin (100 mg/L) and were confirmed by PCR tests.

### Data availability.

The sequences of 18 human-origin and 3 swine-origin *cfr*-carrying plasmids have been deposited in the NCBI GenBank database under BioProject accession number PRJNA841442.
