# Supplementary material for: Spreading of cfr-Carrying Plasmids among Staphylococci from Humans and Animals
Source: Microbiol Spectr. 2022 Nov 22;10(6):e02461-22. doi: 10.1128/spectrum.02461-22 (PMC9769919; doi:10.1128/spectrum.02461-22)
Supplement: Supplemental file 1 — Supplemental material. Download spectrum.02461-22-s0001.pdf, PDF file, 2.7 MB [file spectrum.02461-22-s0001.pdf]

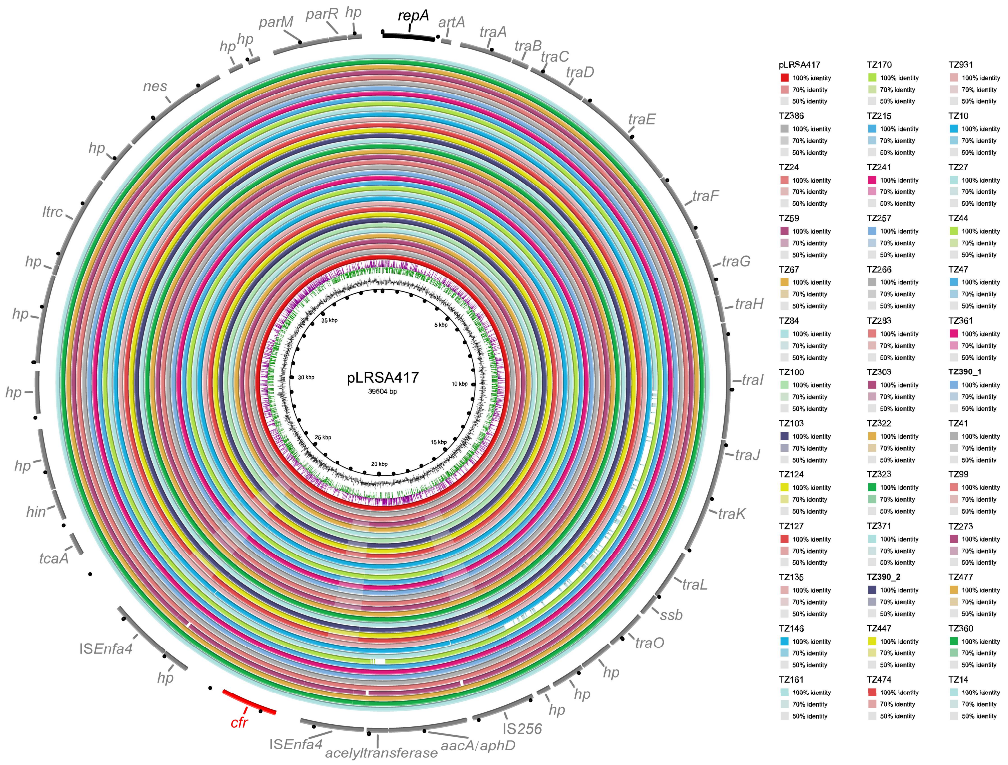

Supplementary figure 1. Alignments of Illumina HiSeq sequences of 38 *cfr*-positive staphylococci of human clinical origin with pLRSA417 plasmid.

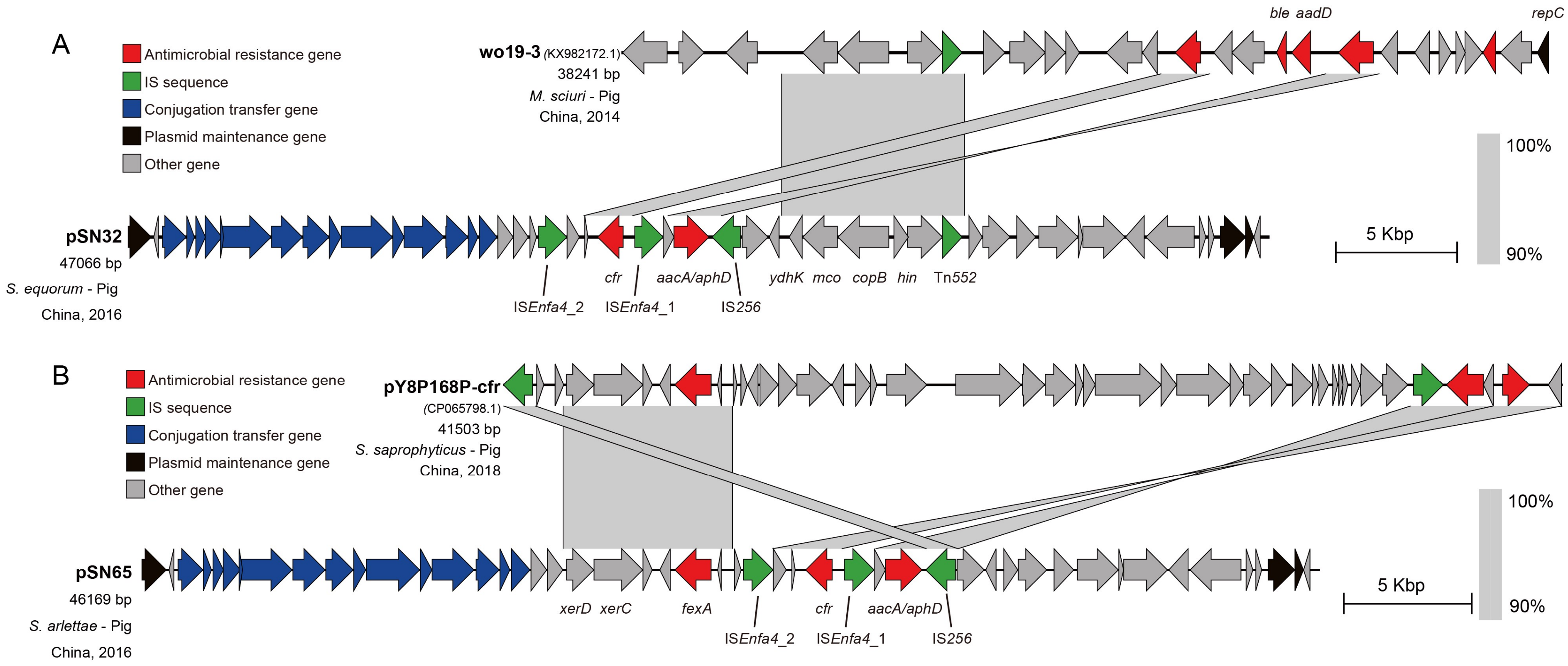

Supplementary figure 2. Sequence alignments of extra sequences in pLRSA417-like plasmids pSN32 and pSN65 of porcine origin.

Supplementary table 1. Sampling information of *Staphylococcus* isolates from human clinical infections.

Species	Total	*cfr*-positive (ratio)
S. aureus	1141	1 (0.09%)
S. haemolyticus	558	4 (0.71%)
Staphylococcus hominis	194	0
S. capitis	113	26 (23.01%)
S. epidermidis	77	0
M. sciuri	53	0
Staphylococcus warneri	42	0
S. saprophyticus	17	0
S. cohnii	14	3 (21.43%)
Staphylococcus caprae	11	0
Staphylococcus lugdunensis	9	0
Staphylococcus simulans	8	0
Staphylococcus gallinarum	6	0
Staphylococcus xylosus	4	0
Staphylococcus auricularis	3	0
Total	2250	34 (1.51%)

Supplementary table 2. Information of *cfr*-positive *Staphylococcus* isolates from human clinical infections.

Strain	Year	Ward[a]	Source	Species
TZ360	2003	BD	Blood	*S. cohnii*
TZ361	2003	BD	Blood	*S. haemolyticus*
TZ386	2004	BD	Blood	*S. capitis*
TZ390_1	2004	BD	Blood	*S. haemolyticus*
TZ14	2011	ICU	Blood	*S. cohnii*
TZ24	2011	ICU	Blood	*S. capitis*
TZ59	2011	ICU	Blood	*S. capitis*
TZ67	2011	ICU	Blood	*S. capitis*
TZ41	2011	IDD	Sputum	*S. aureus*
TZ99	2012	ICU	Blood	*S. haemolyticus*
TZ84	2012	RMD	Blood	*S. capitis*
TZ100	2012	ICU	Blood	*S. capitis*
TZ103	2012	ICU	Blood	*S. capitis*
TZ124	2012	ICU	Blood	*S. capitis*
TZ127	2012	EMD	Blood	*S. capitis*
TZ135	2012	ICU	Blood	*S. capitis*
TZ146	2012	EMD	Blood	*S. capitis*
TZ161	2012	ICU	Blood	*S. capitis*
TZ170	2012	ICU	Blood	*S. capitis*
TZ215	2012	US	Urine	*S. capitis*
TZ241	2012	ICU	Blood	*S. capitis*
TZ257	2012	ICU	Blood	*S. capitis*
TZ266	2012	ICU	Blood	*S. capitis*
TZ283	2012	EICU	Blood	*S. capitis*
TZ303	2012	EICU	Blood	*S. capitis*
TZ322	2012	BD	Blood	*S. capitis*
TZ323	2012	RMD	Blood	*S. capitis*
TZ273	2012	ICU	Blood	*S. cohnii*
TZ477	2013	ICU	Blood	*S. haemolyticus*
TZ371	2013	ICU	Blood	*S. capitis*
TZ390_2	2013	ICU	Blood	*S. capitis*
TZ447	2013	ICU	Blood	*S. capitis*
TZ474	2013	ICU	Blood	*S. capitis*
TZ931	2014	ICU	Blood	*S. capitis*
TZ10	2019	BD	Blood	*S. capitis*
TZ27	2020	BD	Blood	*S. capitis*
TZ44	2021	BD	Blood	*S. capitis*
TZ47	2021	BD	Blood	*S. capitis*

[a] BD, burns department; ICU, intensive care unit; IDD, infectious diseases department; RMD, respiratory medicine department; EMD, emergency medicine department; US, urology surgery; EICU, emergency intensive care unit.

Supplementary table 3. Information of porcine isolates that carry pLRSA417-like plasmids.

Plasmid	Species	Year	Location	Size (bp)
pSN32	*S. equorum*	2016	Henan, China	47066
pSN65	*S. arlettae*	2016	Henan, China	46169
pSN134	*S. saprophyticus*	2015	Shandong, China	46169

Supplementary table 4. Information of pLRSA417-like plasmids from the NCBI GenBank database.

Plasmid	Species	Year	Source	Location	Accession Number
pH29-46	*M. lentus*	2016	Chicken	China	CP059680.1
pcfr-XZ03	*S. capitis*	2019	Human	China	CP077712.1
pLRSA417	*S. aureus*	2013	Human	China	KJ922127.1
pXWZ	*S. capitis*	unknown	Human	China	MT096435.1
pSR01	*S. aureus*	2013	Human	China	CP048644.1
pSX01	*S. xylosus*	unknown	Pig	China	KP890694.1
pSP01	*S. epidermidis*	unknown	Human	Italy	KR230047.1
p12-00322	*S. epidermidis*	2012	Human	Germany	KM521836.1

Supplementary table 5. Locations and sources of *cfr*-carrying staphylococcal sequences from the NCBI GenBank database.

	Human	Animal	Total
Chromosomal DNA	5	8	13
Plasmid	27	30	57
Total	32	38	70
